# On ‘the Politics of Repair Beyond Repair’: Radical Democracy and the Right to Repair Movement

**DOI:** 10.1007/s10551-024-05705-z

**Published:** 2024-05-15

**Authors:** Javier Lloveras, Mario Pansera, Adrian Smith

**Affiliations:** 1 Postgrowth Innovation Lab, Universidade Vigo, Vigo, Spain; 2 Science Policy Research Unit, University of Sussex, Falmer, UK; 3 Postgrowth Innovation Lab, Edificio Benito Corral, Rúa Benito Corral, 45, entrada Javier Puig, 36001 Pontevedra, Spain

**Keywords:** Right to repair, Repair movement, Radical democracy, Hegemony, Planned obsolescence, Assetization, Repair studies, Circular economy, Postgrowth

## Abstract

This paper analyses the right to repair (R2R) movement through the lens of radical democracy, elucidating the opportunities and limitations for advancing a democratic repair ethics against a backdrop of power imbalances and vested interests. We commence our analysis by exploring broader political-economic trends, demonstrating that Original Equipment Manufacturers (OEMs) are increasingly shifting towards asset-based repair strategies. In this landscape, hegemony is preserved not solely through deterrence tactics like planned obsolescence but also by conceding repairability while monopolizing repair and maintenance services. We further argue that the R2R serves as an ‘empty signifier’, whose content is shaped by four counter-hegemonic frames used by the R2R movement: consumer advocacy, environmental sustainability, communitarian values, and creative tinkering. These frames, when viewed through Laclau and Mouffe’s theory of radical democracy, reveal different potentials for sustaining dissent and confronting OEMs' hegemony in the field of repair. Analysed in this way, an emerging business ethics of repair can be understood as driven by *the politics of repair beyond repair.* This notion foregrounds the centrality of non-violent conflict and antagonism for bringing radical democratic principles to repair debates, looking beyond narrow instrumentalist conversations, where repairability is treated as an apolitical arena solely defined by concerns for eco-efficiency and resource productivity.

## Introduction

Original equipment manufacturers (OEMs) have constructed multiple barriers that hinder the ability of independent repairers and consumers to fix their own products (Perzanowski, 2022). The enormous ecological costs of these trends are well documented, with some of the most vulnerable communities in the Global South being disproportionally affected by global e-waste inflows (Forti et al., 2020). Repair barriers and restrictions, however, have wide-ranging implications beyond the proliferation of e-waste. To illustrate some of these ramifications: John Deere has come under public censure for their restrictive repair policies, which lock out independent repair shops and burden small farmers with exorbitant fees and restrictions when seeking repairs for their tractors (Koebler, 2017). Healthcare professionals denounce how repair restrictions cause inefficiencies and delays on vital treatments—e.g., when manufacturers refused to supply hospital technicians with repair manuals and other essential resources to fix broken ventilators during the COVID-19 pandemic (Koebler, 2020). Military personnel are similarly prevented by manufacturers from performing essential repairs on their own equipment, consequently increasing the likelihood of equipment failure, and putting their safety at risk (Ekman, 2019).

Against this backdrop, the right to repair (R2R) movement has attracted a diverse range of supporters, including technology activists, independent repair businesses, and grassroots organizations (Perzanowski, 2022). These actors demand not only more repairable product designs, but also greater access to essential repair resources—e.g. spare parts, specialized tools, repair manuals, diagnostic tools—and the removal of software barriers that hinder third-party and self-repair. Some of these demands are starting to make inroads into policymaking, especially in the United States (US) and the European Union (EU), where R2R regulations have already been implemented and more are currently under discussion (Hernandez et al., 2020; Svensson-Hoglund et al., 2021). At the same time, corporations such as Tesla, John Deere, General Electric, Caterpillar, or eBay, have openly opposed the R2R by citing concerns with costs, design, customer safety, intellectual property, or brand reputation, to name a few. Other companies, such as Apple, have adopted a more ambivalent public stance by taking some steps towards offering self-repair services whilst still lobbying against R2R legislation behind the scenes (Allendorf, 2018; Stone, 2023).

As with other technology-centered controversies arising from users and civil society (Hess, 2007), the R2R movement brings into focus the influence of democratic struggles over complex technological systems (Stirling, 2014). This observation sets the stage for our research, which aims to examine the R2R debate through the lens of radical democracy (Laclau & Mouffe, 2001). Proponents of this approach embrace the role of dissensus as a necessary catalyst to foster radical democratic critique and interventions within business ethics (e.g. Castelló & López-Berzosa, 2023; Couch & Bernacchio, 2020; Kenny & Bushnell, 2020; Vachhani, 2020; Barthol & Bloom, 2020; Fougère & Solitander, 2020; von Redecker & Herzig, 2020; Dawkins, 2015, 2021). Collectively, these studies expose the hegemonic condition of the spaces in which conventional approaches to business ethics are theorized and practiced. They show how the social structures that perpetuate uneven power relations are often masqueraded behind consensus, proposing instead a dissensus-based approach whereby business ethics is treated as both immanent and intrinsic to political struggle—a movement spearheaded by civil society rather than the traditional corporate channels of social responsibility (Rhodes et al., 2020).

Rhodes encapsulates these developments through the notion of ‘democratic business ethics,’ dispelling the traditional view of business ethics as involving a consensus-based, socially disembedded process, and proposing instead an “ethics through which corporations are held responsible not to themselves, but to society” (Rhodes, 2016, p. 1512). Building on this, our research aims to provide a democratic business ethics perspective on the R2R. More specifically, *we aim to elucidate the opportunities and limitations for advancing a radically democratic perspective on repair amid the power imbalances and vested interests underpinning the field*. By doing this, our work contributes to the field of business ethics in the following ways.

First, previous research provides nuanced accounts of situated repair practices in diverse empirical settings, mostly in repair cafés (Meißner, 2021), but also in workplaces and households (e.g., Strebel et al., 2019). Repair has also been studied in connection to consumer communities (Godfrey et al., 2022) and DIY lifestyles (Watson & Shove, 2008). However, several authors have observed that the broader structural trends and power dynamics overbearing these repair practices have received less attention (Graziano & Trogal, 2017, 2019, 2023; McLaren et al., 2020). Our work addresses this gap by showing how OEMs are increasingly adopting asset-based approaches to repair, where hegemonic relations are maintained not only by discouraging repair—e.g., through planned obsolescence (Guiltinan, 2009; Slade, 2006), but also, and arguably most importantly, by conceding repairability while establishing monopolies over repair and maintenance services.

Second, we delve deeper into the intersection of dissensus and business ethics by combining insights from social movements studies with the theory of hegemony and radical democracy developed by Laclau and Mouffe (2001). Thus, in line with previous work on social movements (e.g. Reineke & Ansari, 2016; Valor et al., 2021), we conduct a fine-grained empirical analysis of the collective action frames shaping R2R activism. Based on this, we identify four ways of framing the R2R, namely the consumer advocacy frame, the environmental sustainability frame, the communitarian frame, and the creative tinkering and grassroots innovation frame. However, working abductively (Timmermans & Tavory, 2012), we push forward this analysis by integrating it with the rich theoretical repertoire provided by Laclau and Mouffe’s (2001) theory. Our approach is encapsulated in the notion of *the politics of repair beyond repair*, a term that we coin and apply to our analysis. Through this concept, we offer a valuable metaphor for understanding how the different frames produced by R2R activists intersect with the politics of radical democracy, indicating the centrality of building counter-hegemonic coalitions by incorporating broader societal interests and agendas, while simultaneously highlighting the recognition of the irresolvable tensions and social contradictions which drive the movement’s vitality.

Finally, these reflections have important implications for the circular economy debate, in which repair features as an important technique, and where critics have noted a tendency to privilege technocratic solutions, leaving virtually no space for critique and radical transformation (Murray et al., 2017; Valenzuela & Böhm, 2017). Our research contributes to this critique by showing how R2R activists contest dominant repair discourses, aligning them with a wide range of ethical values and aspirations for greater democratic control over repair systems. These struggles, essential for fostering a wider set of collective freedoms to fix, maintain, and tinker with products, find themselves marginalised by a prevalent portrayal of the R2R as merely another circular economy strategy for boosting repair behavior and rationalising repair efficiencies (e.g. Marikyan and Papagiannidis, 2023; Jin et al., 2023; Hernandez et al., 2020). Our study provides a necessary corrective to this reductionist trend. Limiting the R2R debate to concerns over environmental efficiency and repair behavior is not only empirically flawed, but also perpetuates a conceptual tunnel vision—one which diminishes the scope for present and future business ethics considerations of the R2R.

The paper is structured as follows. First, we present our theoretical framework, followed by a description of our methods. This paves the way to our analysis, which unfolds through multiple layers. It starts by discussing macro level dynamics and how these translate into hegemonic repair discourses employed by OEMs. Next, analysis turns to the R2R movement, identifying the different frames employed by R2R activists. Then, these frames are analysed through the lens of radical democracy, discussing their possibilities and limitations to challenge OEMs’ hegemony in the field of repair. The paper closes by articulating the key contributions, conclusions, and limitations of our study.

## Theoretical Framework

### Framing and Technology and Product-Oriented Movements

The R2R movement can be understood as a Technology and Product-Oriented Movement (TPMs), defined by Hess (2005, p. 517) as movements whose “principal means of social change is the development of new or alternative forms of material culture”. Unlike traditional social movements, which often place technology in the background of more salient social, political, or environmental grievances, TPMs distinctively focus their efforts on the development and modification of products and technological systems, fostering alternative relationships between individuals, their communities, and the sphere of technology. In opening-up innovation decisions to alternative aims, perspectives, and values, TPMs contest the direction of technology and product developments in society and emphasize the political characteristics of ostensibly technical matters. Consistent with this conceptualization, the R2R movement champions new product designs that are more amenable to repair, as well as public access to critical repair resources, from skills and knowledge, to specialized tools, spare parts, and repair software.

The framing activities of TPMs are central to the contestation processes underpinning the social construction of technology (Bijker, 1997). Framing lies at the heart of the semiotic mechanisms whereby activist groups and technology movements seek to infuse alternative values within the content and direction of innovation and technical systems (Smith, 2005). From a social movements’ perspective, Benford and Snow (2002, p. 614) define collective action frames as “action-oriented sets of beliefs and meanings that inspire and legitimate the activities and campaigns of social movement organization”. In this regard, the concept of framing denotes “the signification work” (Reineke & Ansari, 2016, p. 301), which is carried to perform three functions (Benford & Snow, 2000), namely: *diagnostic functions* (e.g., identifying and naming the problem or issue at hand, setting the boundaries of what is considered relevant for discussion, helping people understand why it matters, and who or what might be responsible); *prognostic functions* (e.g., providing solutions to the diagnosed problem, outlining what actions need to be taken and by whom); *motivational functions* (e.g. galvanizing individuals and groups to act by instilling a sense of urgency, moral imperative, or collective identity). Additionally, the ideological orientation underlying collective action frames has implications for the tactical repertoire a movement employs, shaping whether their members opt for radical and confrontational tactics, reformist and collaborative tactics, or a combination of both (Den Hond and De Bakker, 2007).

A corollary is that the success of TPMs—such as the R2R—hinges on their ability to frame their grievances, demands and aspirations through culturally resonant narratives and semiotic systems, which enable them to mobilize stakeholders, convey a sense of urgency, stir emotions, construct legitimacy, garner support, and so forth for their alternative orientations towards technology and products to become influential in social, policy and business change. This approach to framing, conceived as strategically oriented signification work carried out by actors, has been widely employed within the business ethics field, especially in conjunction with institutional theories of legitimacy (e.g., Gutierrez-Huerter et al., 2023). But while useful for unveiling the symbolic, rhetorical, and emotional work of TPMs, an analysis of collective action frames alone offers limited scope to elucidate how struggles over the R2R come to be imbued with political content. We attribute this limitation to the absence of an *explicit* theorization of “the political” within conventional framing analyses. In this paper, we address this omission of the political by supplementing our analysis of TPM collective action frames with insights from radical democracy theorists, specifically those elaborated by Chantal Mouffe and her joint theorization of hegemony with Ernesto Laclau. What follows is not intended to provide a full-fledged discussion and contextualization of radical democracy and its implications for business ethics in general, which can be found elsewhere (see Rhodes, 2016; Rhodes et al., 2020), but to introduce key insights that will be incorporated into our political analysis of repair ethics.

### Radical Democracy and the Struggle for Hegemony

The thrust of radical democracy is to both extend and deepen traditional democratic ideals by fostering political diversity and contestation (Mouffe, 1996, 2005). This requires linking multiple democratic struggles against a common adversary (Laclau & Mouffe, 2001). Conflict is acknowledged as both an inescapable and indispensable component of democracy, a perspective which diverges from deliberative democratic theories that emphasize rational discourse and consensus (Rhodes et al., 2020). Specifically, non-violent conflict assumes a pivotal role in contesting and destabilizing entrenched power hierarchies (and their concomitant discourse), thereby creating spaces for the inclusion and participation of marginalized voices and communities. Undemocratic relations between actors are sustained through hegemony, a concept whose presence is a source of conflict in societies, and which finds its roots in Marxist theory, especially in the work of Antonio Gramsci. Hegemony refers to the way in which dominant classes or groups establish and maintain their privileges, not only through coercion but also through the cultivation of collective norms, ideas, and values that become 'common sense' to subordinate groups. Drawing on the work of Gramsci, Laclau and Mouffe (2001) extend the notion of hegemony beyond the confines of class struggle, encompassing a diverse array of social movements, antagonisms, and undemocratic power relations, from race and gender issues to nationalism.

Focusing on the role of discourse, Laclau and Mouffe (2001, p. x) argue that *hegemony* is asserted when ‘a particular social force assumes the representation of a totality that is radically incommensurable with it’. *Articulation* is the dynamic process through which a particular group brings certain elements—e.g., demands, grievances, aspirations, etc.—into a temporary relation with one another to create what is perceived as a unified and coherent discourse. Within the process of articulation, *chains of equivalence* are the specific linkages or relations established among certain elements. When chains of equivalence are established between social actors, their particularities are momentarily set aside to stress what they have in common. This makes these elements coalesce not only in the pursuit of a shared objective, but also in their opposition to a common antagonist.

However, while a hegemonic social formation may seem to represent a universal interest, their hegemony is always contingent and partial. It represents a particular claim of universality that inevitably creates an outside; that is, an exteriority made by all those interests, demands, or identities that were not articulated into the hegemonic discourse (Laclau, 2005). There is also a *logic of difference* at play, whereby actors resist surrendering their particularities even as they participate in broader hegemonic formations. The incomplete nature of any hegemonic formation, in turn, means that there are always gaps, exclusions, or points of “incommensurability” (Laclau, 2007). But the incompleteness of hegemony alone does not necessarily lead to a counter-hegemonic response from those excluded. In this regard, an important distinction made by Laclau and Mouffe (2001) is between *subordination—*which refers to relationships between actors characterized by power imbalances, and *oppression—*which occurs when uneven power relations are no longer perceived as natural, legitimate, or fair. It is only when marginalized actors begin to perceive their situation as oppressive—rather than subordination—that these relations become a site of antagonism, and by extension, a potential site for radical democracy.

Thus far, we have argued that actors must perceive their relations of subordination as oppression. However, for Laclau and Mouffe (2001), an additional step is necessary to articulate a counter-hegemonic response: namely that the different relations of oppression coexisting within a given field come to be viewed as mutually equivalent. Central to this process is the role of *empty signifiers*, such as “freedom,” “equality,” or “justice,” which serve as versatile rallying points around which disparate demands and values can coalesce owing to the signifiers’ adaptable meaning and broad appeal (Zueva & Fairbrass, 2021). Empty signifiers become the nodal points around which a different logic of equivalence can be set in motion by counter-hegemonic actors (Laclau, 2007).

But while empty signifiers create space for political engagement and contestation, their ambiguity and universal appeal simultaneously offers opportunities for hegemonic forces to re-articulate these same signifiers in ways that consolidate their power over the terrain of popular discourses. In this regard, Laclau (2005, p. 87) acknowledges that empty signifiers can incorporate elements from “entirely different political signs”—which explains why “between left-wing and right-wing populism, there is a nebulous no-man's land which can be crossed – and has been crossed in many directions”. This process of ideological co-optation and neutralization is central to the reproduction of capitalist hegemony (Hamilton & Ramcilovic-Souminen, 2023), and has been shown by business ethics researchers to operate in contexts as diverse as collaborations between NGOs and corporate actors (e.g., Baur & Schmitz, 2012), frugal innovation (Tesfaye & Fougère, 2022), or corporate responses to countercultural consumer movements (Holt, 2002).

Based on the above, we coin ‘the politics of repair beyond repair’ as a key concept to encapsulate and summarize our analytical framework. On the one hand, this notion highlights the potential of repair politics to articulate broad societal aspirations and struggles that transcend the sum of particularistic concerns with product repairability. Simultaneously, the idiom ‘beyond repair’ alludes to an unfixable state, foregrounding “the constitutive role of conflict and antagonism, and the fact that division is irreducible” (Mouffe, 2005, p. 274). In essence, we posit that the R2R operates an empty signifier that embodies ‘the politics of repair beyond repair’ in this dual sense: first, by transcending mere product repairability to address broader societal aspirations, and second, by thriving amid dissensus, and acknowledging the irreparable social antagonisms and conflicts that shape the possibilities for product repair and maintenance in capitalist societies. The subsequent sections will analyze and elaborate on how the “politics of repair beyond repair” operate in practice. However, before delving further, we describe the methods used for this analysis.

## Methodology

This research adopted an abductive logic of inquiry, which involves a recursive process of double-fitting data and theories (Timmermans & Tavory, 2012). This means that the researchers’ theoretical repertoire is interwoven with both data collection and data analysis from the outset (Alvesson & Sköldberg, 2018). Therefore, whilst we present our theory and methodology here through separate sections and sub-sections for clarity purposes, data collection and analysis in abductive research projects typically unfolds simultaneously in “stochastic, highly dynamic and reflexive ways” (Sætre & Van de Ven, 2016, p. 687).

### Data Collection

Fieldwork spanned from September 2021 to November 2023, during which we collected a diverse array of primary and secondary data (see Table 1 for further details). We employed a theoretical sampling strategy, where the selection of empirical materials was driven by their anticipated relevance to develop a theoretical understanding of the R2R movement and its politics, rather than representativeness or generalizability (Charmaz, 2014). The primary data consisted of 25 semi-structured interviews categorized as follows: 15 with repair activists and campaigners, 5 with technicians affiliated with OEMs, 2 with industry representatives, and 3 with civil servants involved in Right to Repair (R2R) legislation. All participants were recruited by utilizing the research team's professional and personal networks, enhanced by snowballing techniques that leveraged the networks of the interviewees. To protect the anonymity of the participants, their names have been replaced by pseudonyms, and any potentially revealing details have been suitably altered or omitted.

**Table 1:** Data collection

Primary data		
Category of interviewees	Interviewee number	Further details
Repair Activists	15	Online semi-structured interviews with 15 repair activists, ranging in age from 25 to 75, including 10 males and 5 females. Each interview lasted 40 to 50 min. Participants were recruited through snowball sampling and had at least 3 years of experience in repair activism and campaigning. Geographically, 6 were based in EU countries, 2 in the UK, 3 in the USA, 1 in Canada, and 1 in Australia
Repair Technicians	5	Online semi-structured interviews, each lasting between 30 and 40 min, with a sample of 5 males, aged 32 to 50. They were recruited through the researchers' professional networks and were employed in the authorized repair networks of major OEMs. All participants had over 4 years of industry experience in the consumer electronics sector
Industry Representatives	2	Online semi-structured interviews with 2 industry representatives (1 male and 1 female), each lasting approximately 30 min. The interviewees, recruited through the researchers' professional networks, held middle-management positions in EU trade associations—in the home appliances and automotive sectors
Policymakers	3	Online semi-structured interviews, each lasting about 30 min, were conducted with three EU Parliament policy advisors involved in R2R legislation. All participants had at least 5 years of experience in relevant policy areas and were recruited through the researchers' professional networks

Secondary data		
Data type	Number of items	Further details
Documents and materials produced by organizations supporting the R2R	140	From September 2021 to November 2023, we gathered a range of materials from organizations instrumental in the Right to Repair movement. Our dataset included blog posts, newsletters, and discussions from community forums. We focused on iFixit (www.ifixit.com); Restart (www.therestartproject.org); Repair EU (www.repaireu.org); Repair Café International (www.repaircafe.org); PIRG (www.pirg.org); and The Repair Association (www.repair.org)
Media Coverage	75	Our data was enriched with English-language media coverage from European and North American outlets, comprising 75 articles, opinion pieces, and reports from September 2017 to November 2023. This media analysis, though not systematic, provided insights into public discourse, policy debates, and the societal impact of the movement
Policy documents and legislative reports	10	We collected various industry reports and policy documents on an ad hoc basis, including white papers, legislative proposals, and guidelines, primarily from the EU and US, to deepen our understanding of the institutional and regulatory landscape surrounding the R2R debate

Interview data were augmented by an array of secondary materials, including blog posts, discussion in online forums, or weekly newsletters from organizations central to the R2R movement, such as iFixit, Restart, Repair EU, Repair Café International, Public Interest Research Group (PIRG), and the Repair Association. Additionally, we utilized non-systematic media coverage to gain contextual insights into public discourse and policy debates. Lastly, we collected industry reports and legislative materials from the EU and the US on an ad hoc basis, further deepening our grasp of the emerging regulatory landscape surrounding R2R. These materials proved valuable to 'cast a wider net' and delve into 'unprecedented features of the context' (Behfar & Okhuysen, 2018, p. 333).

### Data Analysis

Tavory and Timmermans (2014, p. 5) conceive abductive analysis as “a creative inferential process”, where the researcher moves back and forth between theoretical and empirical materials. This sensemaking process, driven by “disciplined imagination” (Sætre & Van de Ven, 2021, p. 690) relies heavily “on the scope and sophistication of the theoretical background a researcher brings to the research” (Timmermans & Tavory, 2012, p. 173). Unlike purely inductive research, which begins with data to generate theories, or deductive research, which tests theories against data, abductive research focuses on the generation of new insights or theoretical accounts that offer plausible explanations for so-called *anomalies—*that is “unexpected phenomenon that cannot be explained or is poorly understood using existing knowledge” (Sætre & Van de Ven, 2021, p. 684).

Abductive analysis integrates elements from both deductive and inductive reasoning, operating through a distinctive iterative process as outlined by Sætre and Van de Ven (2021). As the researcher navigates iteratively between theory and data, the abductive process advances, refining initial insights and progressively shaping them into increasingly nuanced and sophisticated accounts of complex phenomena. At an operational level, data analysis for this project was supported by the software for qualitative data analysis NVivo. We followed an abductive coding approach, which combined recommendations by Vila-Henninger et al. (2024), and Deterding and Waters (2021). The main analytical operations, performed through several abductive cycles/iterations, are depicted below:

#### Generating an Abductive Codebook

This operation involved two types of coding strategies, one deductive and the other inductive (Vila-Henninger et al., 2024). Given the iterative nature of abductive research, the abductive codebook was treated as a ‘living document’ which evolved throughout the entire research process. Initially, we started with deductive codes derived from the literatures, mostly on ‘collective action frames’ and ‘assetization.’ As the analysis progressed, and prompted by reviewers, we incorporated into the codebook additional codes and sub-codes derived from Laclau and Mouffe’s theory of radical democracy—e.g., ‘hegemony’ ‘counter-hegemony’, ‘empty signifier,’ ‘chain of equivalence’ and ‘antagonistic frontier’. Supplementing this, our codebook incorporated categories generated inductively; that is, they emerged from a close reading of the data rather than the literature—e.g., ‘Barriers to repair’, ‘monopoly’, ‘environment’, ‘circular economy’ and ‘degrowth’, ‘grassroots innovation’, ‘hacking’, ‘community’, or ‘consumer advocacy’, were created through inductive coding and incorporated to the codebook at different stages of the research.

#### Performing Abductive Data Reduction Through Indexing and Code Equations

Data reduction is a strategy to focalize the analysis on relevant sub-sets—rather than the whole—dataset. This was achieved first by indexing the data (Deterding & Waters, 2021), and, at a later stage, through code equations (Vila-Henninger et al., 2024). Indexing is the opposite of word-by-word coding, involving the selection of large chunks of text containing cases, events, or quotes, which we found particularly interesting or relevant—triggering what Deterding and Waters (2021, p. 628) dub “aha” moments. As we familiarised ourselves with the data, we began to perform abductive code equations, that is: the creation of codes to account for phenomena that spanned across individual codes (Vila-Henninger et al., 2024, p. 16). Through constant comparisons (Charmaz, 2014), we looked for instances in the data where deductive and inductive codes intersected. For example, data deductively coded as ‘collective action frames’ frequently intersected with many of the inductive codes and sub-codes relating to ‘environment’ (e.g. ‘circular economy’; ‘waste’, ‘degrowth’), ‘community’ (e.g. ‘repair cafés’, ‘sharing’, ‘solidarity’); ‘grassroot innovation’ (e.g. ‘hacking’, ‘makers’; ‘curiosity’), or consumer advocacy (e.g. ‘product ownership’; ‘monopoly’; ‘financial disadvantage’, ‘product quality’). After corroborating these code equations, we established and filled in with content the four collective action frames underpinning the R2R movement—discussed in "Disrupting OEMs’ Hegemony: The R2R Movement and ‘the politics of repair beyond repair’" section.

#### In-depth Abductive Qualitative Analysis

Here, researchers are concerned with a refinement of the codes and making sure that the analytical categories and relations identified are compelling and holistically coherent. In our case, this operation involved revisiting the code equations, eliminating superfluous or overlapping codes, discarding of uncorroborated relations or unsatisfactory lines of inquiry, and further press the interpretation to identify overlooked empirical or conceptual anomalies. For example, during the initial stages of analysis we ascribed significant analytical weight to codes related to ‘planned obsolescence’ or ‘barriers to repair,’ mostly focused on technical issues. Through a more in-depth analysis, however, we gradually realized that these codes had a more limited analytical scope than we initially expected. The opposite happened with ‘radical democracy’ codes, which played a secondary role during the preliminary stages but became central as our analysis progressed. In this regard, the inputs from the three anonymous reviewers contributed to further refine our account of the R2R, as they drew attention to anomalies that eluded our initial explanations, while offering insights and hunches that pushed the analysis forward. This is consistent with Timmermans and Tavory’s (2012) view of abductive analysis as a social rather than an individual process, extending to peer review and other exchanges with members of the research community.

## Contextualizing Hegemony in the Field of Repair

The R2R movement is often depicted in opposition to a series of repair barriers imposed by OEMs, which prevent independent service providers and consumers from repairing their products (e.g., Perzanowski, 2022). Such barriers are diverse and range from design and manufacturing choices that make products virtually unrepairable, to the use of intellectual property law for withholding access to essential repair resources—these barriers are outlined in Table 2 but see also Gordon (2019). Nonetheless, this paper contends that the ethico-political dimension of the R2R movement must be understood as an ideological struggle against hegemony in the interconnected spheres of technology and consumer goods, rather than a piecemeal opposition to concrete repair barriers. Consequently, our first analytical move will be to abstract away from concrete repair restrictions/policies that R2R activists oppose and lay out the broader political–economic context wherein their struggle against hegemony unfolds.

**Table 2:** OEM’s main barriers and strategies to restrict DIY and independent repair

Area of concern	Description	Examples
Design and manufacturing	Devices are designed in ways that make them harder to open and service, such as the use of glued, fused, or soldered components, non-removable batteries, and nonstandard/proprietary screws	Earbuds cannot be opened without damaging them (Dixit, 2023)
Restrictive proprietary software and digital locks	Smart devices depend on proprietary software, and access to software updates, patches, and support can be restricted. OEMs also use copyright laws, user license agreements (EULAs), firmware, encryption, and access controls to enforce digital locks and control repair and diagnostic software	John Deere Tractors (Koebler, 2017)
Part pairing strategies	OEMs use serialized parts that must be paired with devices by specialized proprietary software, making it harder for third parties to perform repairs	Apple is using part pairing across their product range (Greenlee, 2023)
Withholding access to repair manuals	OEMs use trademarks, copyrights, or patent laws to withhold access to repair manuals and schematics	Medical equipment manufacturers (Koebler, 2020)
Limiting availability of original parts	OEMs establish captive markets for original spare parts and components, limiting their availability for independent and DIY repair	Nikkon (Chamberlain, 2012) or Tesla (Heilweil, 2022)
Exploiting legal loopholes and customer ignorance on consumer rights	OEMs use stickers and other threats of voiding the warranty to discourage DIY repair	Harley Davidson (Robertson, 2022)
Psychological obsolescence	Customers are discouraged from repairing products that are no longer viewed as stylish or desirable and are incentivized to trade in old models for new ones	Most consumer gadgets follow these trends (Perzanowski, 2022)

### From Planned Obsolescence to Assetization

Historically, critics have explained the proliferation of repair barriers and restrictions as manifestations of planned obsolescence (e.g., Perzanowski, 2022; Slade, 2006). Planned obsolescence refers to corporate strategies that intentionally shorten a product's lifespan to encourage replacement purchases by consumers (for different taxonomies of planned obsolescence see Rivera & Lallmahomed, 2016; Cooper, 2010; Guiltinan, 2009). With advanced capitalist economies developing towards a perennial state of overproduction, it makes little economic sense for OEMs to prioritize easy-to-repair products over strategies that emphasize shorter product cycles, frequent repurchases, and disposability (Slade, 2006). This trend is most acute in the affluent consumer societies of the Global North, where consumer markets for smartphones, flat-screen TVs, laptop computers and so forth are mature, and OEMs must release new models at shorter intervals to compete for customers’ attention and spending. Easy-to-repair products run counter to these dynamics since they encourage longer-term ownership and product longevity rather than product replacement. Furthermore, products designed for repairability are typically more expensive and inferior in terms of design and/or performance, making them vulnerable to competition from the influx of ever cheaper, lighter, thinner, and sleeker alternatives (Cooper, 2004, 2005).

However, while planned obsolescence offers a plausible explanation for some restrictions on independent and autonomous repair, it does not satisfactorily account for all of them. Consider, for example, John Deere's use of digital locks to prevent third-party repairs. It is somewhat obvious that these policies are not implemented to make farmers replace their tractors more often. The same holds true for many high-value consumer products, capital goods, and professional equipment, from cars to advanced medical devices, to industrial machinery, where transaction costs are significant, and buyers tend to prioritize performance over novelty factors. Companies in these sectors impose severe restrictions on essential repair resources (e.g., repair software, information, specialized tools, skills, and replacement parts), but they do not do so with the intention of rendering their products obsolete. We argue that a different explanation, set against the backdrop of a transforming political economy, is necessary to fully understand the scope of these phenomena.

One such explanation lies in the shift from commodities to assets. Birch and Muniesa (2020) highlight that the asset form is displacing the commodity in advanced capitalist economies. The term “asset” refers to any tangible or intangible resource that enables asset-owners to generate a recurring income stream (Birch, 2017). Although assets are tradable like any other commodity, their true value lies in an ability to capture rents over the long term rather than being limited to one-time transactions (Birch & Muniesa, 2020). OEMs pursue assetization through building different forms and bundles of product–services (Baines & Longfoot, 2013), downplaying product ownership for access, and tethering consumers to multiple post-sales offerings (Hoofnagle et al., 2019). In business-to-business contexts, for example, new contractual arrangements such as leasing, renting, licensing, and subscription-based models are promoting new market relations based on rentiership rather than ownership. Following this trend, for example, Rolls-Royce, Airbus, and Boeing are becoming providers of engine operating services; that is leasing their engines and providing repair, maintenance, and overhaul services, with airlines paying a set fee per flight hour.

In business-to-consumer contexts, companies are also assetizing their product lines by constructing complex ecosystems of services around them. Here, the customer may retain ownership of the product but pays a subscription fee to access key services, without which the product's utility would diminish. For example, PlayStation owners are unable to use their devices to play multiplayer games online unless they subscribe to PlayStation Plus. Similarly, Apple's revenues are increasingly driven by subscriptions to services including Apple Care, Apple Music, Apple TV+, iCloud. In any case, assetization enables OEMs to overcome market saturation by deemphasizing the need to artificially boost product replacement rates and instead focusing on extracting rents from an expanding array of services bundled with their devices.

Extant research suggests that the market for repair services in the US and Europe is increasingly concentrated in the hands of OEMs, while independent repair shops continue to decline in number (Svenson-Hoglund et al., 2021). This trend is consistent with OEM’s transition from planned obsolescence to asset-based accumulation regimes, where the formation of repair monopolies is important for several reasons. At the most fundamental level is the question of market power. OEMs aim at maximum control throughout a product’s lifecycle because, in the context of assetization, physical products are a gateway to controlling the expanding market for intangible product–services. In this regard, highly controlled devices enable companies to lock-in users to their own service ecosystems both technically and contractually (Hoofnagle et al., 2019), whereas allowing independent repair, modification, and tinkering erodes these entry barriers and “provides a foothold for the competition from which to grow and expand its product and/or service offerings” (Baines & Longfoot, 2013, p. 93).

Asserting control over repair also presents an opportunity for OEMs to create alternative revenue streams around their existing product base, capturing an income that would otherwise go to third parties (e.g., independent repairers, a spare part supplier, etc.). They typically do this through the creation of networks of authorized repair shops, which acquire from the OEM an official license to repair and service their products (Warren & Gibson, 2021). In return, OEMs impose a franchise fee and, more importantly, become providers of authorized spare parts and components, tools, repair manuals and software, as well as for the ongoing staff training and certifications that franchisees require to maintain their license. In more extreme cases, companies such as Nikon or Tesla bypass any authorized intermediaries and deliver repair and maintenance services directly to their customers. This exclusivity enables OEMs to obtain higher profit margins for repair and maintenance services, but it also poses significant strain for customers in terms of waiting times, costs, and convenience (Heilweil, 2022).

Furthermore, by providing repair and maintenance services, OEMs cultivate ongoing relationships with customers, building trust and loyalty over time. This can help increase customer retention and reduce customer acquisition costs. It also places OEMs in a more favorable position to upsell and cross-sell additional product–services, including upgrades, extended warranty, insurance, additional accessories, or other supplementary products or services. Finally, having control over the repair process enables companies to collect vital information and data about their product–assets, such as usage trends, failures, and maintenance requirements. This information can be used to improve product–asset design, production, and marketing, or even sold to third parties, which further reinforces the assetization of their products.

Therefore, although assetization has its own logic within capitalism, and this logic cannot be directly attributed to R2R pressures, an added advantage for OEMs is that they reassert their hegemonic power, and see off other stakeholders, by expanding control over products and aftermarket services such as repair, maintenance, and upgrading (Hoofnagle et al., 2019; Warren & Gibson, 2021).

### Articulating a Hegemonic Repair Discourse

To be able to operate across planned obsolescence and assetization, OEMs must produce a hegemonic moral and symbolic order in which repair restrictions appear as natural, inevitable, and representing the general interest. In the context of planned obsolescence, this is attained by coupling OEM’s resistance to produce more durable and repairable devices with a series of ostensibly pro-consumer and business virtues, such as demand responsiveness, customer satisfaction and market competitiveness. Within this hegemonic articulation, promoting more repairable products is viewed as impractical insofar as it would impose unreasonable trade-offs—e.g. price, weight, thinness, performance vs. repairability—which not only threaten to undermine OEM’s competitive position, but also run counter to their customers’ interests. Here, OEMs are positioned as mere intermediaries between consumers and their allegedly “natural” desires for ever faster, thinner, and more affordable devices. Thus, their opposition to pro-repair regulation masquerades as a consumer orientation. A representative from the European home appliances industry, interviewed for this study, illustrates this perspective:

Consumer demand drives us to continually innovate. Our customers expect devices that are lightweight, cost-effective, and cutting-edge. Balancing these demands with the additional requirement of making all products easily repairable would compromise the very qualities our customers seek (…) Many design choices that right to repair advocates label as ‘planned obsolescence’ or ‘anti-repair’ are, in fact, reflections of our commitment to deliver the highest possible value to our consumers (Carl, 51, Industry representative).

However, as argued in the previous section, restrictive repair strategies are increasingly oriented towards enabling asset-based forms of accumulation rather than planned obsolescence. The purpose is to enable OEMs to further control and monetize their products through aftermarket services like maintenance, insurance, repair, and upgrade. Here, OEMs depend on a different hegemonic articulation; one where repair is construed as a profit-driven technical service, designed exclusively to extend product–service lifecycles, and mitigate waste at a profit. With repairability decisions being reduced to cost–benefit calculations, any alternative values, motivations, and forms of relating to repair—e.g. the idea of repair as a vehicle for self-expression, autonomy, or intellectual inquiry—are relegated to the periphery, framed as eccentricities that deviate from the ‘common sense’ of economic rationality. Within this hegemonic repair discourse, demands for increasing public access to essential repair resources—such as specialized tools, manuals, diagnostic software, or spare parts—appear as irrational, since the primary concern shifts to the cost-effectiveness of repairing, upgrading, or replacing a product, irrespectively of who holds the control and power over the process.

Moreover, independent, and autonomous repair options are depicted as risky, unprofessional, or illegitimate by hegemonic assetization discourses. That is, repairing products outside OEMs and their authorized networks, is constructed as a high-risk endeavor which exposes lay consumers to all sorts of jeopardy—e.g. substandard services and damage to their devices, risks associated with counterfeit components, hacking and data theft, loss of warranty or even physical harm through malfunction of improperly repaired devices. By creating a climate of fear and distrust around independent repair practices, OEM's restrictive repair strategies are presented as being in the interests of consumers, not against them. These articulations can be illustrated with a couple of examples, drawn from major trade associations. For instance, APPLIA, the main trade association of home appliance manufacturers in Europe states that “consumers not only have a right to repair, but most importantly a right to have their products repaired right. If an appliance is not properly repaired, safety within the home could be compromised” (APPLIA website, available here: https://www.applia-europe.eu/press-releases-applia/promoting-sustainable-consumption-through-repair). Similarly, the Advanced Medical Technology Association (AMTA), which represent manufacturers of medical equipment in the US, states that:

The “right to repair” complex medical devices is wrong for patients (…) Proponents of the so-called “Right to Repair” movement demand that unregulated third-party servicers be given unlimited access to service manuals and other proprietary OEM information. Such a move would only serve to put patients and device users at greater risk. Access to the latest manuals is no substitution for the extensive training, knowledge and expertise provided by the OEM (AMTA website, available here: https://www.advamed.org/our-work/key-issues/R2R-wrong-for-patients/).

Finally, the widespread acceptance of OEMs’ repair restrictions is often achieved by their alignment with the interests of society more widely—not only consumers. Here restricting repair is argued as essential to protect intellectual property, and this, in turn, is argued as necessary to protect innovation. In other words, repair restrictions are depicted as good for society because they provide OEMs with incentives to continue to bring innovative products to market. This chain of equivalence is nicely illustrated by the following quote from the Wall Street Journal’s editorial on the R2R:

American innovation is dependent on the protection of intellectual property. It encourages innovation by discouraging theft. But there are those who are philosophically opposed to intellectual property protection. Left-leaning public interest law firms and activist groups (…) are pushing their anti-innovation agenda in the guise of a right to repair (Giovanetti, 2021: NP)

## Disrupting OEMs’ Hegemony: The R2R Movement and ‘the politics of repair beyond repair’

Thus far, we have illustrated how calls for greater freedom for fixing, maintaining, and tinkering with products outside OEMs’ authorized repair networks, clash with the hegemonic discourses surrounding repair. When calls for increasing repair options outside OEMs and their authorized networks are evaluated through the prism of hegemony, these calls clash with the established common sense, and as a result they appear as unreasonable, eccentric, impractical, or even illegitimate. R2R activists face the task of articulating a counter-hegemonic formation, establishing chains of equivalence that would connect a diverse array of interests, identities, and values that are marginalized by hegemonic assetization discourses and repair practices. A key challenge, however, lies in the highly heterogenous nature of such a constituency, spanning DIY aficionados, farmers, military personnel, consumer advocacy groups, pro-repair businesses such as Fairphone, Framework or Backmarket, independent repair shops, healthcare professionals, community and grassroot repairers, environmentalists, technology enthusiasts, so-called makers and hackers, and others.

Drawing on Laclau and Mouffe (2001), we argue that the “rights” in the R2R operate as an empty signifier—a versatile rallying point whose ambiguity creates a discursive space wherein disparate interests, values, and identities, can be realigned against a common adversary. In this regard, the advocacy of “rights” possesses a strong cultural resonance and almost universal appeal, especially in Western contexts, but at the same time, the ambiguity and malleability of the term allows for actors to promote the R2R from multiple positions—e.g. from radical civil rights struggles to conservative and liberal traditions. As an empty signifier, the R2R can play this dual role. On the one hand, the R2R stands for a universal aspiration that transcends the disparate interests behind the movement. On the other hand, the R2R’s meaning remains inherently contingent and open to negotiation, allowing for a variety of particularistic demands, meanings, and identities to be articulated around it.

Moreover, by mobilizing the notion of ‘rights’ as an empty signifier, the R2R movement taps into a broader, culturally resonant discourse that is deeply ingrained in public consciousness and legal structures, without foreclosing the possibility of diverse interpretations. Collective action frames contribute to the dynamic interpretive work that fleshes out, refines, and adapts the R2R signifier to various constituencies and their respective interests and identities. Our analysis finds four collective action frames through which the R2R is articulated, namely: *the consumer advocacy frame, the environmental sustainability frame*, *the communitarian frame*, and *the creative tinkering and grassroots innovation frame.*

### The Consumer Advocacy Frame

R2R advocates often frame their aims in terms of consumer advocacy, emphasizing the protection of consumer rights and welfare in the face of OEM restrictive repair policies. Framed this way, ‘the right to repair is about bringing power back to consumers and is founded on concepts of utilitarianism and consumer autonomy’ (Montello, 2020, p. 184); and thus, by extension, “opposition to the ‘right to repair’ is anti-consumer, short-termism and stark exploitation” (Singh, 2023: NP).

This R2R frame asserts that the notion that consumer rights extends beyond the point of purchase, depicting repair restrictions as a violation of consumers' right to own and control their products fully. In this regard, for example, iFixit states that: “The Right to Repair movement is founded on a fundamental principle: If you bought it, you own it, and you should be able to fix it” (iFixit, available here: https://www.ifixit.com/Right-to-Repair#repair-is-freedom). Yet, this frame's resonance and impact are markedly enhanced when it positions the R2R not only as a consumer right to be protected from OEMs, but also as integral to advancing consumer welfare. Within this context, advocates of the R2R argue that OEMs’ restrictions on self-repair and independent repair services not only contravene fundamental consumer rights but also detrimentally impacts consumer welfare. By limiting repair options, OEMs curtail competition in the repair market, leading to higher repair costs and promoting a cycle of frequent, unnecessary product replacements (Hanley et al., 2020). R2R demands are thus positioned as essential for safeguarding consumer welfare, offering consumers financial relief and broadening their choices in the marketplace.

These articulations are instrumental in establishing a chain of equivalence that extends beyond niche audiences, enabling the R2R agenda to resonate with the immediate concerns and experiences of mainstream consumers. Indeed, this frame enables the R2R movement to strategically link their demands to consumers’ self-interest and garner broad support, as illustrated by one of our interviewees:

Oh, for me, it’s all about getting real value for the money I spend. I mean, why should I be shelling out hundreds more when a simple fix could extend my phone's life by another year or two, eh? The Right to Repair is about having that choice, you know? It saves us money, sure, but it also makes us smarter consumers. I talk about it this way to my friends, and they immediately get it. They see how it directly affects their wallets (Mel, 28, R2R activist in Canada).

R2R activists mobilize a consumer advocacy frame to establish alliances with consumer organizations and seek reforms to consumer rights institutions. Simultaneously, this framing enables them to strategically position R2R demands within the broader common-sense prevailing in capitalist societies—where core values such as consumers’ self-interest, market competition, ownership, or consumer sovereignty and freedom of choice, are widely endorsed and constitute the dominant discourse. Therefore, rather than articulating the R2R as a radical discourse, which could alienate conservative and mainstream audiences, this R2R frame projects a moderate and pragmatic consumer agenda aimed at reforming existing market institutions to enhance fairness, welfare, and competition.

### The Environmental Sustainability Frame

Another prominent R2R frame is oriented towards environmental sustainability, and the protection of environmental rights. In this regard, R2R advocates draw attention to how restrictive repair policies contribute to environmental problems such as growing waste, intensifying mineral extraction and climate change by reducing the lifespan of products and encouraging faster replacement rates among consumers. OEM’s repair barriers are fundamentally regarded as manifestations of a linear extract-make-use-dispose economy, and a throwaway consumer culture, both of which are increasingly untenable in a future faced with constrained resource availability and access (e.g., European Commission, 2020).

On this basis, the R2R is portrayed as a key circular economy strategy aimed at reducing waste, improving resource conservation, and mitigating greenhouse gas emissions (Hernández et al., 2020). This is complemented by an equally strong emphasis on the economic benefits of R2R policies such as expanding repair markets, creating jobs, boosting local economies, supporting small businesses, and encouraging more innovative product designs and circular business models (e.g., European Commission, 2020). This framing is also prevalent in academic accounts of the R2R. In this regard, for example, Marikyan and Papagiannidis (2023, NP) state that “the broader goal of the right-to-repair regulation is to address the environmental challenges by ensuring social and economic growth without compromising on natural resources”.

Therefore, the environmental sustainability frame typically portrays the R2R as a market-friendly, green growth-driven agenda, closely linked with the principles of the circular economy. However, our analysis also identifies more radical articulations that recognize the existence of environmental limits to growth—in line with post growth and degrowth principles (Bradley & Persson, 2022). In this regard, some activists view the R2R not as a circular economy strategy to pursue growth by greener means, but as part of a deeper socio-economic transformation to escape the cycle of ever-increasing production and consumption. When observing that the postgrowth R2R articulations are overshadowed by a focus on the circular economy, our interviewees interpreted this differential emphasis in strategic terms:

(…) in our Right to Repair circle, many of us see a clear connection to the circular economy—spurring the creation of jobs and businesses in the repair sector, reducing waste, it's clear-cut. A smaller number entertain degrowth, but that’s a deeper dive. Talking circular economy is a smoother approach; it's about green jobs and waste, which easily clicks with people, as opposed to the profound degrowth angle (Daniel, 45, R2R activist in Spain).

Therefore, while providing an opening to radical politics—an aspect absent in the consumer advocacy frame—the environmental sustainability frame of the R2R is predominantly articulated through mainstream circular economy tropes and concerns.

### The Communitarian Frame

Given important developments in R2R took root in repair cafés and online networks of repair enthusiasts, it is unsurprising that an important framing of purpose within the movement is conceived as a community-empowering idea, where emphasis is placed on the relational and transformative aspects of repair practices (e.g., Bradley & Persson, 2022; Meißner, 2021). From this perspective, technology–society relations have historically evolved in ways that alienate communities, and one way in which such alienation takes place is by rendering people unable to fix their products. In this vein, the International Repair Cafés Network states in their website that “the trouble is, lots of people have forgotten that they can repair things themselves. Especially younger generations no longer know how to do that. Knowing how to make repairs is a skill quickly lost” (www.repaircafe.org).

Therefore, by taking part in collective acts of repair and mending, individuals can connect with others and restore their collective agency in relation to technology. For example, one of the main coordinators of the European R2R campaign, the Restart Project, states as one of their strategic aims that ‘we will continue to frame repair as a social activity, taking away the fear and potential downside of repair by making it about human connection’ (https://therestartproject.org/about/strategy/).

Fixing things outside the market for professional repair services is thus viewed as an opportunity for individuals to share tools, skills, and knowledge with one another, building a sense of community, solidarity, and collaboration, and to anticipate more convivial productive activity with technology, rather than competitive and individualistic acquisition (Strebel et al., 2019). OEMs’ strategies for planned obsolescence and restrictive control of repair are perceived by this R2R frame as an enclosure of a fundamental right to enable commons-based and collaborative repair ecosystems to flourish (Zapata Campos et al., 2020). Thus, based on notions of community resilience, solidarity and technical empowerment, this frame constructs the R2R as a collective right to the communal access to and non-market distribution of essential repair resources—e.g., knowledge, skills, tools, or spare parts. This is not to say that other goals such as reducing waste are treated as unimportant within the communitarian frame, but salience is always in relation to shared benefits of empowerment, care, socialization, quality of life, or social inclusion (Bradley & Persson, 2022).

### The Creative Tinkering and Grassroots Innovation Frame

Lastly, the R2R is framed as part of an ongoing struggle to unlock the full potential for technological creativity and innovation in society, identifying OEMs' restrictions on DIY repair and product modification as major roadblocks in this pursuit. It starts from the view of repair as ‘a vital source of variation, improvisation and innovation’ (Graham & Thrift, 2007, p. 6), where the aim of the repairer is not necessarily limited by a desire to restore objects to their original state. On the contrary, repair is viewed as ‘an important engine by which technological difference is produced and fit is accomplished’ (Jackson, 2014, p. 227). Repair is thus seen as a productive and creative generator of economic resilience and wellbeing and as such a component within grassroots innovation movements (Smith et al., 2017).

When framed in these terms, the potential of the R2R is illustrated through examples of how giving people the freedom to fix, upgrade, tinker with products catalyzes individuals’ disposition towards innovation and creativity to the benefit of society:

Although some may argue that Right-to Repair laws are bad for business and stifle innovation, it’s hard to ignore the success of the Open-Source Software and Maker Movements, which both assert that users have the right to fix or modify any product they legally own. The fruits of these movements, including the Linux and Android operating systems, the Arduino and Raspberry Pi computing platforms, and the RepRap 3D printer project, are an integral part of many of the products and services that continue to fuel our economy’s current wave of innovation and prosperity (Goldberg, 2018: NP).

Here the rationale for a R2R is justified in the view that OEMs’ restrictions on DIY repair and tinkering represent a hindrance to the human potential for innovation and creativity. In this regard, for example, they point to the COVID-19 pandemic, when citizens utilized their skills and resources to make and donate open-source ventilators, masks, and protective gear by using 3-D printing technology at a time when many OEMs treated the shortage of life-saving equipment in hospitals as an opportunity to increase profit margins (e.g., Richterich, 2020).

However, while our findings foreground a juxtaposition of multiple R2R framings, it is important to acknowledge a hierarchy among them. Notably, the consumer advocacy and the circular economy frames occupy central positions in the R2R discourse, highlighting a strategic logic grounded in cultural and policy resonance. In global terms, the US and EU are spearheading R2R policy and regulation. In the US, the R2R movement is primarily perceived as a market issue, focusing on consumer rights and competitive practices. This perception is underscored by the pivotal role the Federal Trade Commission plays in regulating R2R claims and related consumer advocacy (see Federal Trade Commission, 2021). Within this scenario, frames of consumer advocacy dovetail with emergent concerns regarding the evolving landscape of intellectual property and product ownership in the digital economy (Perzanowski & Schultz, 2016), as well as with long-standing apprehensions regarding excessive corporate power and a perceived erosion of consumer sovereignty in the marketplace (Perzanowski, 2022). Meanwhile, in the EU, consumer protection is also crucial, but the R2R is primarily viewed in environmental policy terms. Accordingly, the circular economy offers the R2R a consolidated policy frame to articulate itself within the EU’s green growth industrial strategy—e.g., the R2R is explicitly referred to in the EU’s Circular Economy Action Plan (European Commission, 2020).

### Towards a Counter-hegemonic Articulation of Repair

Framing processes precipitate a threefold politicizing effect, which we unpack below through the lens of radical democracy. Collectively, this threefold politicization in R2R serves to dislocate the entrenched corporate narrative surrounding repair, creating a rupture in the hegemonic fabric. Firstly, as counter-hegemonic practices subvert the idea that restrictive repair policies incarnate the only reasonable, practical, and legitimate approach, extant relations of subordination unfolding within the field of repair begin to be perceived in terms of oppression—where the uneven dynamics of control and access to essential repair resources are no longer rationalized or presented as natural. When this occurs, OEMs’ consensus over the rightful limits of repair, often settled through technocratic approaches such as cost–benefit analyses, is replaced by conflict and antagonism, which is a condition for democratic business ethics (Rhodes, 2016). These antagonisms unfold through a range of anti-hegemonic tactics employed by the R2R movement, which we summarize in Table 3.

**Table 3:** Linkages between R2R framings and their counter-hegemonic tactics

R2R movement’s repertoire of actions	Examples	Primary framings of repair	Secondary framings of repair
Grassroot events (e.g., repair cafés, repair clinics, library tools)	Repaircafe.org Restart parties Fixit clinics	Communitarian	Environmental sustainability
Advocacy and lobbying	The Repair Association—US (www.repair.org) Right to Repair Europe—EU (www.repair.eu)	Consumer advocacy (in the US) and environmental sustainability (in the EU)	Environmental sustainability (in the US) and consumer advocacy (in the EU)
Litigation	Lawsuits against Kodak, Harley Davidson, or John Deere	Consumer advocacy	Environmental sustainability
Media exposés	Media exposé of John Deere or Apple’s anti-repair policies	Consumer advocacy	Environmental sustainability and creative tinkering
Education and training	iFixit’s wiki style repository of repair manuals, teardowns, and tutorials	Creative tinkering	Consumer advocacy
Partnerships with businesses	iFixit’s partnerships with Microsoft, Motorola, or HTC	Environmental sustainability	Consumer advocacy

Second, building on Laclau (2007), the tactical and action repertoire of the R2R movement contributes to the formation of an ‘antagonistic frontier’, a divide which separates the interests of OEMs from what can be broadly termed as ‘the people’. This antagonistic frontier, more than a mere division, becomes a politically charged site of identity formation, attesting that “there cannot be radical politics without the definition of an adversary” (Laclau & Mouffe, 2001, p. xvii). In this regard, it is important to acknowledge that R2R advocates originate from highly heterogeneous concerns and backgrounds. For example, conservative voting U.S. farmers seeking economic self-sufficiency may, on first inspection, find little common ground with DIY enthusiasts driven by a quest for commons-based repair. Healthcare professionals, focused on patient safety and regulatory compliance, may not naturally resonate with the grassroots ethos of hackers and modifiers of technologies. Similarly, environmental activists, prioritizing sustainability, may diverge from independent repair shops operating under market-driven imperatives. Rather than cementing their unity on traditionally recognized structural logics such as class or political ideology, the web of solidarities and affinities underpinning the R2R movement are sustained by their opposition to OEMs. For illustrative purposes, consider the following statement from iFixit, a leading R2R organization:

People are having trouble getting the repair parts and information they need for tractors, appliances, wheelchairs, ventilators, hearing aids, snowmobiles, boats—the list goes on. If it can be fixed, some company has probably tried to create a repair monopoly on it (iFixit website, available here https://www.ifixit.com/Right-to-Repair#repair-is-freedom).

Here, iFixit mobilizes a chain of equivalence between diverse collectives of users, each represented by the reference to specific products—–e.g., farmers (‘tractors’), disabled people (‘wheelchairs’, ‘hearing aids’), households (‘appliances’), or healthcare professionals and their patients (‘ventilators’), articulating their shared interests with the R2R in contraposition to OEMs and repair monopolies.

Third, but relatedly, R2R delineates a new discursive terrain wherein the meaning of the R2R signifier transcends its original attachment to repair-specific concerns and demands, to become equivalent with broader societal interests, in this case representing the advocacy for consumer rights, environmental sustainability, open technological innovation, and community resilience. For example, Ranni, a US based repair activist interviewed for this project stated: *Honestly, fixing my own gear is just the start. Right to Repair? It's bigger than that. It's about us taking back control, you know? Not just over our tech, but over how we treat the planet and how we stand up for our rights as consumers.* And similarly, Miguel, a Spanish repair activist highlighted: *The Right to Repair, for me, is about advocating for a world where technology is open, where we can innovate, create, and not be boxed in by restrictions (…) It's about keeping the true spirit of technological progress alive.* This transfiguration of the R2R into a signifier for broader social causes and aspirations is subtle but crucial, as it mirrors the core tenet of counter-hegemony in Laclau and Mouffe’s (2001) radical democratic theory: that is, the articulatory process whereby a signifier outgrows its initial particularity to represent a new discursive totality.

Thus, our analysis finds the R2R producing a radical democratic politics of repair, challenging OEMs’ hegemony in the repair field through a conflict-based rather than consensual approach (Rhodes et al., 2020). Looking ahead, however, the assetization dynamics identified in "From Planned Obsolescence to Assetization" section could eventually weaken some of the antagonisms driving the movement, especially those articulated through consumer advocacy and circular economy framings. Crucially, asset-based forms of accumulation do not require OEMs to discourage repair and shorten product lifespans. On the contrary, they seek to attain full control over repair and maintenance services as part of a wider set of business practices that leverage their products to establish monopolistic services for continual rent extraction.

Since assetization strategies are fully compatible with the promotion of some product repairability and maintenance, the door is opened for OEMs to either neutralize or re-articulate appropriable R2R demands around consumer advocacy back into hegemonic repair discourses, practices, and policies. For example, whilst OEMs may neutralize some R2R demands by confronting consumers with utilitarian trade-offs (e.g., stylish and affordable vs. easily repairable products), they may also leverage their market dominance and economies of scale to provide additional benefits that independent repair shops may struggle to compete with, such as extended product warranties, bundled post-sales services and product care packages, or loyalty schemes including aggressive discounts. Additionally, a single focus on consumer rights and protection within asset arrangements may help reinforce the contemporary consumerist ethos, potentially neglecting broader concerns related to social equity, environmental sustainability, or community resilience (Graziano & Trogal, 2017, 2023).

The same neutralization threat applies to the *circular economy framing*, where waste minimization and resource efficiency are approached as opportunities to generate new income streams for OEMs (Valenzuela & Böhm, 2017; McLaren et al., 2020). This perspective, which is already integrated in EU policy debates on the R2R, could lead to the co-optation of R2R environmental goals by corporate interests, as repair becomes another avenue for firms to extract value from consumers and consolidate their market power. A related drawback of circular economy articulations is their tendency to reduce the scope of the R2R to narrow questions of resource optimization and operational efficiency. Although boosting repair rates is certainly important, the R2R cannot be reduced to an instrumentalist pursuit of efficiency at the expense of other issues. In this regard, it is crucial to recognize that the R2R encompasses broader concerns about the uneven distribution of power in the repair sector, and how such power inequalities may translate into a loss of autonomy, resilience, community, and technological agency, which transcend circular economy articulations revolving around the productivity and efficiency of repair systems.

A radically democratic perspective therefore seeks to sustain the R2R as a politicizing movement whose meaning is renewed by a constant displacement, rather than dissolution, of the antagonistic frontier—a *war of position* in Gramscian terms. Here is where we believe that the less dominant R2R frames, namely postgrowth sustainability, community, and grassroots innovation, provide solid foundations to sustain a radical democratic approach to repair in the longer term. Indeed, whereas the antagonizing potential of the consumer advocacy and circular economy framings of the R2R is likely to diminish in the future, increasingly veering towards consensus-positions and compromises with OEMs, these alternative R2R frames are primed to foster a vibrant antagonism, challenging and contesting the expansion of asset-based forms of repair in the longer term.

For example, the post growth framing calls into question the very foundations of growth-oriented capitalism. This perspective aligns the R2R with the cultivation of post-capitalist repair relations (Smith, 2023), and the promotion of radical democracy in terms of alternative modes of production and consumption that prioritize ecological sustainability, social equity, and localized, democratic control over resources (Lloveras & Quinn, 2017; Lloveras et al., 2020). Thus, contrary to the circular economy, a postgrowth framing of the R2R draws attention to the risks of uncritically endorsing repairability without first asking fundamental questions about what it is socially useful to produce in the first place and what is harmful and must be scaled back; how much repair would be enough within that new production system, and for what purposes; or how could the value generated by repair labor be better distributed to reduce inequalities and build a fairer and more just society? In this vein, we argue that a postgrowth framing on the R2R must incorporate *the right not to repair*. That is, recognizing that since not all manufactured goods and infrastructures would be compatible with a postgrowth transition (e.g., SUVs, airplanes, oil rigs, transnational pipelines), the individual R2R must be balanced with a collective right to decommission or simply allow certain objects to decay.

Moving on to the *communitarian framing*, this approach may counter the alienating effects of assetization by emphasizing the collective and social aspects of repair, such as the importance of cultivating shared skills and knowledge, as well as fostering a sense of belonging and collaboration (Udall, 2019; Wackman & Knight, 2020). As assetization processes intensify, they could potentially limit opportunities for convivial repair, pushing it underground. In fact, the alienating experiences of individuals entangled in complex asset-based products and services might become a catalyst for renewed social mobilization, directing aspirations towards more convivial relationships with technology. Within this context, the R2R movement could mobilize the communitarian frame to promote subversive, care-based repair practices that challenge the monopolization of repair services and resist the enclosure of commons-based and collaborative repair ecosystems.

Finally, the creative tinkering and grassroots innovation framing, which views repair as a vital source of innovation, creativity, and resilience, may also provide a valuable counterpoint to the assetization strategy. We have shown that assetization runs against the spontaneous drive to engage with technology through tinkering and creative adaptations, a restriction which is justified by OEMs on the grounds of intellectual property, health and safety, and brand reputation. However, the strength of this R2R frame has increased in the context of recent crises, such as COVID, where the capacity of monopolistic repair regimes to respond to emergencies has proven too slow and inadequate. In this regard, the creative tinkering perspective can be mobilized to demonstrate how DIY repair and tinkering activities can contribute to broader societal resilience and innovativeness, such as the development of appropriate technologies, products, and services that address pressing social and environmental aspirations.

## Discussion and Contributions

A focus on dissensus and radical democracy emphasizes the ethical sphere as an arena of non-violent conflict and contestation, where the cultivation of dissensus, rather than consensus, is recognized as a crucial element in fostering democratic processes and challenging entrenched power hierarchies (Rhodes, 2016; Rhodes et al., 2020). In this context, our research extends the current understanding of repair politics and ethics in a number of ways. First, our work directly addresses Graziano and Trogal’s (2017, p. 636) call for “further reflections on [the repair movement's] positioning in relation to the broader societal and political issues in which it is inevitably implicated”. Past research has predominantly illuminated how OEMs restrict repair to encourage premature obsolescence and rapid product replacement (Guiltinan, 2009; Slade, 2006). Our work, however, uncovers a significant but previously overlooked trend: the assetization of repair. This trend diverges from the discouragement of repair seen in planned obsolescence strategies. Instead, assetization involves OEMs establishing monopolies over essential repair resources and services, thereby creating new revenue streams and intensifying control over products and customers.

Our findings help us explain some of the ethico-political tensions and ambiguities observed in the context of repair cafés (e.g., Madon, 2022), workplaces (Strebel et al., 2019), consumer communities (Godfrey et al., 2022), and DIY lifestyles (Watson & Shove, 2008). In this regard, prior research shows that grassroots repair initiatives often default to a service-oriented approach to repair (Madon, 2022), as they are seen by participants not so much as instruments of active resistance against capitalist markets, but rather as means to participate in and/or alleviate their marginalization from these systems (Graziano & Trogal, 2023). Building upon these insights, our work situates the ethico-political tensions observed in grassroots repair initiatives within the larger context of a struggle for hegemony in the repair sector. We show that, in their turn to assetization, the hegemonic practices of OEMs seek to constitute a dominant social logic where repair is understood through a specific set of meanings—profitability, convenience, safety, efficiency, quality, and the necessity of upgrades. In other words, attaining hegemony is not just about economic control but also about shaping how society perceives the act of repair itself, making alternative repair practices, demands, and motivations seem marginal or illegitimate unless they assimilate elements of the hegemonic discourse. It is only through the articulation of a broad-based coalition that counter-hegemonic actors may, over time, be able to redefine the discursive playing field. We have argued that the R2R movement is opening such a political space, but, as we have also shown, this is far from given. Our work paves the way for future research seeking to further integrate micro and macro dimensions, offering conceptual tools for revealing the dynamics of power, dissension, and co-optation between OEMs and repair communities.

Second, our research demonstrates the analytical potential of integrating insights from radical democracy theories with the more conventional framing analyses carried out in social movements literature. In this regard, the study of collective action frames has emerged as a fruitful analytical framework to examine the role of activists as ‘skillful rhetoricians’ (Valor et al., 2021, p. 638), whose discursive work is strategically oriented to persuade audiences and garner their support, trigger emotions, or mobilize publics into action. This is attested by a growing number of business ethics studies mobilizing the notion of collective action frames to deliver fine-grained empirical analyses of the rhetorical and strategic dimensions of social movements discourse (e.g., Reineke & Ansari, 2016; Gutierrez-Huerter et al., 2023). Yet, in the absence of an explicit theorization of “the political,” analyses of collective action frames alone offer limited scope to interrogate framing activities in business ethics at a deeper level. Our work offers a way forward by integrating this analysis with a rich theoretical repertoire derived from radical democratic politics. In doing this, we show that the significance of framing is no longer merely rhetorical and probes instead the ethico-political dimensions of framing activities.

This political layer of analysis is distilled through the notion of *the politics of repair beyond repair*, a new concept encapsulating two interconnected aspects of the R2R movement grounded in Laclau and Mouffe’s (2001) theory of hegemony and radical democracy. First, the politics of repair beyond repair shows how narrow demands for product repairability soon transcend their technical scope, evolving into versatile frames that can articulate repair concerns with broader political agendas, including the promotion of consumer rights, environmental rights, communitarian rights, and the right to repair and tinker. In this first sense, the phrase ‘beyond repair’ denotes this capacity to foster counter-hegemonic politics beyond the specific technicalities and particularities of repair, by weaving together previously dispersed struggles in wider society and bringing them into the field of repair.

At the same time, the phrase ‘beyond repair’ in English also conveys irreparability. In this second regard, our study illuminates how seeking consensus over how repair systems ought to be organized is not necessarily a possible, or even desirable goal, whenever it suppresses underlying conflicts that are pivotal to promote greater democratic control over repair, maintenance, and production in society. Cultivating dissensus is an essential strategy for the future survival and success of the R2R movement as a radically democratic project, and although temporary compromises and ‘fixes’ with OEMs may serve as tactical maneuvers in the short-term (e.g., Apple's recent self-repair program), the R2R movement must remain vigilant against risks of co-optation, as well as that of a gradual dilution of an antagonistic frontier that demarcates ‘us’ from ‘them’.

Our third key contribution confronts the prevalent perspective in business and management literature, which tends to view the R2R merely as a means to boost repair rates, typically in relation to circular economy discourses. This particular focus is evident in recent studies examining the R2R debate from diverse perspectives, including consumer behavior (Marikyan & Papagiannidis, 2023; Roskladka et al., 2023), OEM strategies (Jin et al., 2023), and broader institutional and policy discussions (Hernandez et al., 2020). But while increasing the volume of and frequency of repairs is undeniably important for enabling circular economy strategies, key questions about the (un)fair, (un)democratic, and (un)just design of repair systems, as well as the social inequalities that they may engender, are being superseded by a narrow focus on the rationalization and optimization of repair assets.

Through ‘the politics of repair beyond repair,’ we propose an alternative, more politically oriented pathway to countervail the prevailing line of inquiry focused on behavioral, technical, and legal aspects. Accordingly, the essence of the R2R resides primarily in its potential to radically democratize repair systems, creating agonistic spaces for contesting hegemony. Such conceptualization confronts OEMs and policymakers with new ethical questions revolving around *what should be repaired (and what shouldn’t), who should have the right to repair it,* and *under what institutional conditions can this right be democratically exercised?* These questions matter to business ethics scholars for two reasons. First, because they push current ethico-political thinking beyond a focus on economic incentives, technical efficiencies, and behavioral outputs, to draw attention to a plethora of societal ramifications stemming from the unchallenged acceptance of hegemonic co-option of repair—e.g., the monopolistic power and structure of repair and maintenance systems, the limits of intellectual property in the context of repair, the impact of repair restrictions on consumers’ wellbeing and welfare, the erosion of community resilience and alienation of users, and so on.

Second, approaching the R2R movement as a radically democratic project matters to business ethics because, as we have shown, the meaning of the R2R—as an empty signifier—is inseparable from the struggles of different communities to attain greater democratic control over the repair (and production) systems that they depend on. This fundamental insight is key to “decentring [repair] ethics from business ethics” (Rhodes et al., 2020, p. 628: Our emphasis), widening the scope for exploring the R2R as an evolving discourse capable of furthering multiple, and potentially more radical set of societal aspirations. These include—but are not limited to—the reclamation of technological agency and community empowerment (e.g., Bradley & Persson, 2022), the facilitation of postgrowth (e.g., Barca, 2023) and other post-capitalist modes of organizing consumption and production (e.g., Smith, 2023), or the right to innovate and experiment with technology through the practice of grassroots forms of repair and tinkering (Smith et al., 2017).

## Concluding Remarks

As the recognition of repair grows in capitalist societies, we hope that the nascent business ethics conversation surrounding the R2R will increasingly turn to examining who benefits and who loses from the pressing reconfiguration and expansion of existing repair arrangements, and which demands and agendas are being privileged at the expense of others. Our research is an intervention to steer future debates on the R2R in this underexplored direction. However, that said, it will be vital that further research broadens these considerations beyond the North America and Western European settings. Because of its origin and evolution, the R2R movement has been shaped by debates and discourses around technology that have their special validity in the context of Western consumer culture. Yet, the R2R movement is not the sole site of struggles over technology maintenance and repair. Other forms of ‘technological resistance’ exist in the Global South, and whilst movements have concerns about autonomy and appropriateness that might be recognized in some of the framings identified in our study (and thus scope for articulation), the incumbent ‘politics of repair beyond repair’ will be very different in these other places (e.g., Pansera & Owen, 2018). More research is therefore needed to understand the implications of our analysis in these distinct realities, thereby expanding our perspective beyond the Global North.

Finally, this study reveals how dissensus is more complex, nuanced, and contradictory than simply being in a state of confrontation with a clearly identified opponent. In this regard, the R2R is more than a set of discursive practices and frames for mobilizing actors on one side of an agonistic frontier, whilst identifying a hegemonic opponent on the other. At the same time, it is important to remember that R2R is a TPM that builds alternative material culture through practical projects and activity. Hacks shared online, repair cafes, makerspaces, independent repairers, community stores of spare parts, etc., are, indeed, positive manifestations of dissensus—an alternative material culture—that nevertheless requires continuing and constructive negotiation with the goods and services of OEMs to put the R2R into practical effect. Future research could look deeper into questions about whether and how the material dimensions implicated in the performance of practical repair activities—rather than discourse—compels the R2R movement to pursue a different kind of alliance-building and (democratic) political negotiation compared to the chains of equivalence identified in our analysis. How does the socially rooted activity of demonstrating new material cultures mediate these tendencies? Research needs to consider the co-existence and consequences of such practical-material and political-agonistic strategies and how they can genuinely reinvigorate democracy in business ethics.

## Data Availability

The data supporting this study's findings are restricted to protect participant confidentiality. Access to the data is available from the corresponding author upon reasonable request, subject to an ethics approval process.
